# Associations between insulin action and integrity of brain microstructure differ with familial longevity and with age

**DOI:** 10.3389/fnagi.2015.00092

**Published:** 2015-05-28

**Authors:** Abimbola A. Akintola, Annette van den Berg, Mark A. van Buchem, Steffy W. Jansen, Eline P. Slagboom, Rudi G. Westendorp, Jeroen van der Grond, Diana van Heemst

**Affiliations:** ^1^Department of Gerontology and Geriatrics, Leiden University Medical CentreLeiden, Netherlands; ^2^Department of Radiology, Leiden University Medical CentreLeiden, Netherlands; ^3^Netherlands Consortium for Healthy AgeingLeiden, Netherlands; ^4^Leiden Institute for Brain and Cognition, Leiden UniversityLeiden, Netherlands; ^5^Department of Molecular Epidemiology, Leiden University Medical CentreLeiden, Netherlands; ^6^Department of Public Health, University of CopenhagenCopenhagen, Denmark

**Keywords:** brain, glucose, insulin, Magnetic Resonance Imaging (MRI), Magnetization Transfer Imaging (MTI), age, familial longevity

## Abstract

Impaired glucose metabolism and type 2 diabetes have been associated with cognitive decline, dementia, and with structural and functional brain features. However, it is unclear whether these associations differ in individuals that differ in familial longevity or age. Here, we investigated the association between parameters of glucose metabolism and microstructural brain integrity in offspring of long-lived families (“offspring”) and controls; and age categories thereof. From the Leiden Longevity Study (LLS), 132 participants underwent an oral glucose tolerance test (OGTT) to assess glycemia [fasted glucose and glucose area-under-the-curve (AUC)], insulin resistance [fasted insulin, AUC_insulin_, and homeostatic model assessment of insulin resistance (HOMA-IR)], and pancreatic Beta cell secretory capacity (insulinogenic index). 3 Tesla MRI and Magnetization Transfer (MT) imaging MT-ratio (MTR) peak-height was used to quantify differences in microstructural brain parenchymal tissue homogeneity that remain invisible on conventional MRI. Analyses were performed in offspring and age-matched controls, with and without stratification for age. In the full offspring group only, reduced MTR peak-height in gray and white matter was inversely associated with AUC_insulin_, fasted insulin, HOMA-IR and insulinogenic-index (all *p* < 0.01). When dichotomized for age (≤65 years and >65 years): in younger controls, significantly stronger inverse associations were observed between MTR peak-height and fasted glucose, AUC_glucose_, fasted insulin, AUC_insulin_ and HOMA-IR in gray matter; and for AUC_glucose_, fasted insulin and HOMA-IR in white matter (all *P*-interaction < 0.05). Although the strength of the associations tended to attenuate with age in the offspring group, the difference between age groups was not statistically significant. Thus, associations between impaired insulin action and reduced microstructural brain parenchymal tissue homogeneity were stronger in offspring compared to controls, and seemed to diminish with age.

## Introduction

There is increasing epidemiological evidence that metabolic disorders, including type 2 diabetes (T2D) and its risk factors, such as metabolic syndrome, are associated with risk of cognitive decline and dementia, and with structural and functional brain defects (Burns et al., 2012). T2D has been associated with white matter lesions (Devisser et al., 2011), atrophy (Araki et al., 1994; den Heijer et al., 2003), infarcts (Vermeer et al., 2003), cognitive impairment, and risk of neurodegenerative diseases. Also in the absence of T2D, impaired glucose regulation and higher serum insulin concentrations were found to increase the risk of cognitive decline (Stolk et al., 1997; Yaffe et al., 2006). Likewise, metabolic syndrome was found to be associated with cognitive impairment in middle-aged subjects (Wolf et al., 2007). There are however indications that in old age, the association between cognitive decline and metabolic syndrome, or its individual components, notably obesity and impaired glucose metabolism may be absent or even reverse (van den Berg et al., 2007).

Previously, the Leiden Longevity Study (LLS) (Schoenmaker et al., 2006) was set up to investigate factors associated with familial longevity. We recruited offspring of long-lived nonagenarian siblings, who are predisposed to become long-lived as well, and their partners as controls. We demonstrated that the propensity for longevity in the offspring of these families is marked by preserved insulin sensitivity and a lower prevalence of T2D, compared to controls of similar chronological age (Westendorp et al., 2009; Wijsman et al., 2011). In this relatively healthy and cognitively intact middle-aged to elderly population (Stijntjes et al., 2013), we recently found that metabolic syndrome was specifically associated with microstructural loss of homogeneity of brain parenchymal tissue [assessed by magnetization transfer ratio (MTR) histogram peak height], but not with macrostructural brain damage (Sala et al., 2014). It is unknown whether specific measures of glucose metabolism also associate with microstructural brain parenchymal tissue homogeneity and whether these associations are similar in offspring and controls.

In the current study of 132 participants, we aimed to investigate the association between parameters of glucose metabolism and microstructural brain parenchymal tissue homogeneity in offspring of long-lived families and controls; and with age. Parameters of glucose metabolism were derived from 2-h oral glucose tolerance test (OGTT), and included fasted glucose and area under the glucose curve (AUC_glucose_), which are measures of glycemia; fasted insulin, area under the insulin curve (AUC_insulin_), and HOMA index of insulin resistance, which are measures of insulin resistance; and insulinogenic index which is a measure of pancreatic Beta cell secretory capacity. Microstructural brain parenchymal tissue homogeneity was assessed using magnetization transfer imaging (MTI), which is an advanced, sensitive MRI technique that quantitatively measures microstructural brain parenchymal abnormalities, including reductions in myelin content and in axonal numbers (Filippi and Rocca, 2007) even in brain tissue that appears normal on conventional MR imaging.

## Materials and methods

### Ethics statement

The Medical Ethical Committee of Leiden University Medical Centre approved this study. All participants gave written informed consent to be included in the study.

### Study subjects

Subjects were recruited from the Leiden Longevity Study (LLS), a family based study that was set up to identify genetic factors and biomarkers of familial longevity (Schoenmaker et al., 2006). The LLS consisted of 1671 offspring of Caucasian, nonagenarian siblings (aged older than 89 years for men and 91 years for women, and having at least one sister or a brother fulfilling the same age criteria). Also, 744 of the offspring's partners were included as controls. All eligible subjects were included without selection on health or demographic characteristics.

Of the 2415 subjects in the offspring/partner group, a random subset of 370 subjects underwent MRI scans of the brain and another random subset of 234 subjects underwent an oral glucose tolerance test (OGTT) (Rozing et al., 2010). A total of 132 subjects without diabetes, and with complete datasets for OGTT, 3 Tesla brain MRI and MTI were included for the analysis performed in this study. Furthermore, the participants underwent three cognitive tests-Stroop test, Digit Symbol Substitution Test (DSST) and 15-Picture Word Learning Test (15-PLT). Stroop test and DSST were used to evaluate attention and processing speed, while 15-PLT was used for memory function. Outcome parameter for Stroop test was defined as the time needed to complete the test, while outcome parameter for the DSST was the number of correct digit-symbol combinations within 90 s. For 15-PLT, 15 pictures were successively presented at a rate of one per 2 s after which the subject was asked to recall as many pictures as possible. This procedure was carried out three times (PLT1, PLT2, and PLT3). After 20 min, delayed recall was tested. Outcome parameters were the number of correct pictures after each trial (PLT-immediate) and after 20 min (PLT-delayed) (Stijntjes et al., 2013).

### Oral glucose tolerance test

In the morning after a 10-h overnight fast, fasted glucose and insulin levels were measured, after which subjects ingested a solution containing 75 g anhydrous glucose in 5 min. Thereafter, venous blood samples were collected at 30, 60, and 120 min for determination of plasma glucose and insulin. Areas under the curves (AUCs) obtained in the OGTT were calculated using the trapezoid formula (Pruessner et al., 2003). Homeostatic model assessment of insulin resistance (HOMA-IR) was calculated by the product of the fasting insulin level (mU/L) and the fasting glucose level (mmol/L) divided by 22.5. Insulogenic index was calculated by dividing increments of insulin at 30 min compared to fasting insulin values by the corresponding increment of 30 min glucose levels compared to fasted glucose values.

### Biochemical analysis

Plasma and serum aliquots were frozen at −80°C. All serum measurements were performed using fully automated equipment. Glucose levels were measured using Hitachi Modular P 800 from Roche (Almere, the Netherlands), with coefficient of variation (CV) for measurement less than 5%. Insulin levels were measured using the Immulite 2500 from DPC (Los Angeles, CA). CV was less than 8%.

## Brain MRI protocol

### Image acquisition

Subjects underwent imaging on a whole body MR system operating at field strength of 3 Tesla (Philips Medical Systems, Best, the Netherlands). 3D T1-weighted, T2-weighted FLAIR (fluid attenuated inversion recovery), T2^*^-weighted images, and MTI images were acquired. The dimensions of the images have been previously described in detail (Altmann-Schneider et al., 2013). Image processing and analysis was done using the analytical techniques and tools of the Functional MRI of the Brain (FMRIB) Software Library (FSL).

### Brain volumes

Gray and white matter volumes were calculated using FSL-tool Structural Image Evaluation Normalization of Atrophy (SIENAX). From the whole head input data, brain and skull images were extracted via SIENAX and affine registered to MNI (Montreal Neurological Institute) 152 (Jenkinson et al., 2002). A volumetric scale factor was thus obtained for normalization of head size, after which total brain tissue volume with separate estimates of gray and white matter were obtained.

## Magnetization transfer imaging (MTI)

### Definitions: MTI

MTI is based on interactions between immobile protons (macromolecular protons, probably contained in the cell walls) and free protons of tissue. Mean magnetization transfer ratio (MTR) reflects the average MTR value per structure. MTR peak location reflects the most common MTR value. The peak height of the MTR histogram indicates the number of voxels, which show the most common MTR value per structure, and is considered to be a measure of uniformity of the underlying voxels.

### MTI protocol

Raw magnetization transfer scans were split into M0-sequence (without saturation pulse) and the M1-sequence (acquired after application of a saturation pulse). Brain masks for white matter and cortical gray matter were created using FAST (FMRIB's automated segmentation tool) (Zhang et al., 2001) and FIRST (FMRIB's integrated segmentation tool) on 3D T1-weighted images. To correct for possible partial volume effects, an eroded mask of these segmentations was created by removing one voxel in-plane for all mentioned volumes-of-interest (VOIs) (van den Bogaard et al., 2013). Then, the 3D T1-weighted images were registered to the M0 image using FMRIB's registration tool (FLIRT), and the transformation matrix of this registration was used to register all brain masks to the MTI volumes. Afterwards, individual MTR maps were calculated voxel by voxel following the equation MTR = (M0–M1)/M0. MTR histograms were generated for each VOI. Mean MTR as well as MTR peak height, normalized for the size of the VOI, and MTR peak location were calculated. All MTI measures below −3 or above 3 standard deviations were excluded from statistical analysis.

VBM (Voxel Based Morphometry) analysis was used to study the focal differences and spatial distribution of the changes in gray matter (GM). GM partial volume MTR maps were aligned to MNI152 standard space using the non-linear registration tool FNIRT (FMRIB's non-linear image registration tool). To improve the quality of normalization, averaging the obtained registered GM MTR maps of all subjects created a study-specific MTR template. The native individual MTR maps were then non-linearly re-registered to this template, divided by the Jacobian of the warp field and smoothed with an isotropic Gaussian kernel with a sigma of 3 mm.

## Statistical analysis

Distributions of continuous variables were examined for normality, logarithmically transformed when appropriate, and used in calculations. Median [interquartile range (25th, 75th percentile)] was reported for raw values of variables that were eventually logarithmically transformed (fasted insulin, AUC_insulin_, HOMA-IR and insulogenic index). Differences in sex, smoking status, hypertension, cerebrovascular accident (CVA), myocardial infarction (MI), and use of lipid lowering drugs between the different groups were calculated using Pearson Chi-Square (χ 2) test. Differences in age, BMI, glucose related characteristics, and MRI related characteristics were calculated using independent samples *T*-test for offspring/partner differences. Correlation between cognitive tests and gray and white matter MTR peak height was assessed using bivariate Pearson correlation analysis.

*Z*-values were calculated for standardization of variables. The relation between markers of glucose metabolism and brain structures was determined using linear regression and univariate analysis of variance, and presented as standardized Betas with corresponding *p*-values. Homogeneity of variance assumption was tested using Levene's test. Statistical significance was set as *p* < 0.05. Analyses were performed in offspring and controls before and after stratification of offspring and controls into 2 groups based on age (≤ 65 years, > 65 years). Initial analyses were adjusted for age and sex. Extended models further included smoking status, BMI, use of anti-hypertensive drugs, and use of lipid lowering agents. To check for the effect sizes and interaction between the offspring and controls, an interaction term was added to the model while correcting for covariates. Similar analyses were performed to compare association between OGTT parameters and MRI markers of brain microstructure between age groups.

For statistical analyses, Statistical Package for Social Sciences (SPSS) software for windows (version 20.0) was used. Forest plots were made using GraphPad Prism version 5 (GraphPad, San Diego, CA).

For MRI data, the FSL randomize tool was used to perform permutation-based non-parametric testing (*n* = 5000 permutations) for voxel-wise statistical analyses of the MTR data. Threshold-Free Cluster Enhancement (TFCE) was applied to correct for multiple comparisons. Significance was set at a TFCE corrected *p* < 0.05.

## Results

The current study was performed in a subgroup of 132 participants of the LLS, with the aim of investigating the association between OGTT derived parameters and brain integrity in groups that differ in familial longevity and in age. After measurement of fasted glucose and insulin levels, participants underwent a 2-h oral glucose tolerance test (OGTT). Parameters derived from the OGTT included measures of glycemia (fasted glucose and AUC_glucose_); measures of insulin resistance (fasted insulin, AUC_insulin_, and HOMA index of insulin resistance); and a measure of pancreatic Beta cell secretory capacity (insulinogenic index). Brain volumes were measured using MRI. Furthermore, Magnetization Transfer Ratio (MTR) histogram peak height (henceforth referred to as MTR peak height) was measured using magnetization transfer imaging (MTI). MTR peak height provides an estimate of microstructural brain parenchymal homogeneity, with lower MTR peak height being indicative of loss of homogeneity of the brain tissue. Characteristics of the study subjects are presented in Table 1, for the full study sample and stratified for offspring and controls. The study population from which both OGTT data as well as MTR and MTI data were available (*N* = 132) consisted of 47% males, with mean age of 66.2 ± 6.6 years. Mean BMI was 26.4 ± 3.9 and mean fasted glucose was 5.08 mmol/L. Characteristics were similar between offspring (*N* = 75) and controls (*N* = 57), except for use of lipid lowering drugs, fasted glucose, and AUC_glucose_, which were significantly higher in the partner group.

**Table 1:** **Characteristics of study subjects**.

	**Whole group**	**Familial longevity**	
		**Offspring**	**Controls**
**Number of participants**	**132**	**75**	**57**
**DEMOGRAPHICS**			
Age in years (range)	66.2 (49–84)	66 (52–84)	66 (49–81)
Men, n (%)	62 (47)	43 (57)	27 (47)
BMI in kg/m2	26.4 (3.9)	26.6 (4.2)	26.3 (3.6)
Current smoking, n (%)	11 (8.3)	4 (5)	7 (12)
**MEDICAL HISTORY**			
Hypertension, n (%)	29 (22)	15 (20)	14 (25)
CVA, n (%)	1 (0.8)	1 (1.3)	0 (0)
Myocardial infarct, n (%)	1 (0.8)	0 (0)	1 (1.8)
Lipid lowering medication use, n (%)	14 (10.6)	4 (5) ^*^	10 (18)^*^
**GLUCOSE RELATED CHARACTERISTICS**			
Fasted glucose in mmol/L	5.08 (0.6)	4.97 (0.5)^*^	5.21 (0.6)^*^
AUC glucose	13.93 (3.5)	13 (3)^*^	15 (4)^*^
Fasted insulin in pmol/L, median (IQR)	7 (4, 10)	7.0 (4.0, 10.0)	7.0 (3.5, 10.5)
AUC Insulin, median (IQR)	93.7 (64, 140)	93.7 (65, 139)	91.8 (60, 155)
Insulinogenic index, median (IQR)	13.7 (8, 20)	15 (9, 20)	10 (7, 18)
HOMA-IR index, median (IQR)	1.5 (0.9, 2.2)	1.4 (0.9, 2.2)	1.6 (0.8, 2.5)
**BRAIN VOLUMES (cm^3^**)			
White matter	699 (38)	694 (41)	705 (34)
Gray matter	702 (40)	702 (36)	702 (45)
**MTR PEAK HEIGHT, PIXEL COUNT × 10^3^**			
White matter	117 (24)	118 (25)	116 (23)
Gray matter	74.3 (1.2)	75 (13)	73 (10)
**COGNITIVE TESTS**			
DSST, correct answers	46.46 (9.5)	46.0 (13.2)	46.33 (12.4)
Stroop test, seconds	47.95 (12.5)	48.41 (13.2)	48.26 (11.4)
15-PLTi, correct pictures	10.38 (1.9)	10.27 (1.9)	10.54 (1.9)
15-PLTd, correct pictures	11.35 (2.0)	11.51 (2.1)	11.08 (1.9)

*Unless otherwise stated, values are means (standard deviation). Age refers to age at MRI examination. Brain volumes are normalized for skull size. BMI, body mass index; CVA, cerebrovascular accident; DSST, digit symbol substitution test; AUC, area under the curve; IQR, interquartile range (25th, 75th percentile); 15-PLTi, 15-Picture Word Learning Test-immediate recall; 15-PLTd, 15-Picture Word Learning Test-delayed recall*.

* *p-value < 0.05*.

Also, the outcomes of the cognitive tests were not significantly different in offspring and controls (Table 1). Furthermore, from the cross-sectional data, no significant correlation was found between the cognitive tests, which are a measure of functional brain integrity, and MTR peak height, which is a measure microstructural brain parenchymal tissue homogeneity, either in the whole group (*n* = 132) or in offspring and controls (Supplementary Table S1).

### Familial longevity: markers of glucose metabolism and microstructural brain parenchymal tissue homogeneity

To investigate the association between markers of glucose metabolism and microstructural brain parenchymal tissue homogeneity (assessed using MTR peak height) in individuals that differ in familial longevity, analyses were done in the offspring of long-lived families and controls (Table 2). In the offspring, decreased gray matter (GM) MTR peak height was significantly associated with higher indices of reduced insulin action [fasted insulin (*p* = 0.007), AUC_insulin_ (*p* < 0.001), HOMA-IR (*p* = 0.007)] and OGTT-derived measure of pancreatic Beta cell secretory capacity [insulinogenic-index (*p* < 0.001)]. Similar results were obtained for white matter. In the controls, similar trends were seen but the effects were smaller and mostly did not reach statistical significance.

**Table 2:** **Association of MTR peak height with markers of glucose metabolism in offspring and controls**.

	**Offspring**		**Controls**		***P*_interaction_**
	**Beta**	***p*-value**	**Beta**	***p*-value**	
**GRAY MATTER**					
Fasted glucose	−0.166	0.289	−0.135	0.140	0.856
AUC glucose	−0.062	0.693	−0.145	0.119	0.733
Fasted insulin	−0.378	**0.007**	−0.079	0.384	0.070
AUC insulin	−0.473	**<0.001**	−0.107	0.245	0.021
HOMA-IR index	−0.382	**0.007**	−0.091	0.314	0.083
Insulinogenic index	−0.383	**<0.001**	−0.106	0.374	0.101
**WHITE MATTER**					
Fasted glucose	−0.104	0.486	−0.014	0.896	0.745
AUC glucose	−0.097	0.518	−0.157	0.147	0.691
Fasted insulin	−0.371	**0.005**	−0.050	0.638	0.082
AUC insulin	−0.430	**0.001**	−0.129	0.225	0.086
HOMA-IR index	−0.366	**0.006**	−0.047	0.658	0.091
Insulinogenic index	−0.294	**0.005**	−0.038	0.785	0.150

*MTR histogram height from magnetization transfer MRI was used as a measure of microstructural brain parenchymal tissue homogeneity. All insulin values were log-transformed. Associations are from linear regression analysis, corrected for age and sex, and are presented as standardized Beta coefficients (per increase in SD) with corresponding P-values*.

*The bold values represent significant results (p-values < 0.05)*.

**Figure 1:**
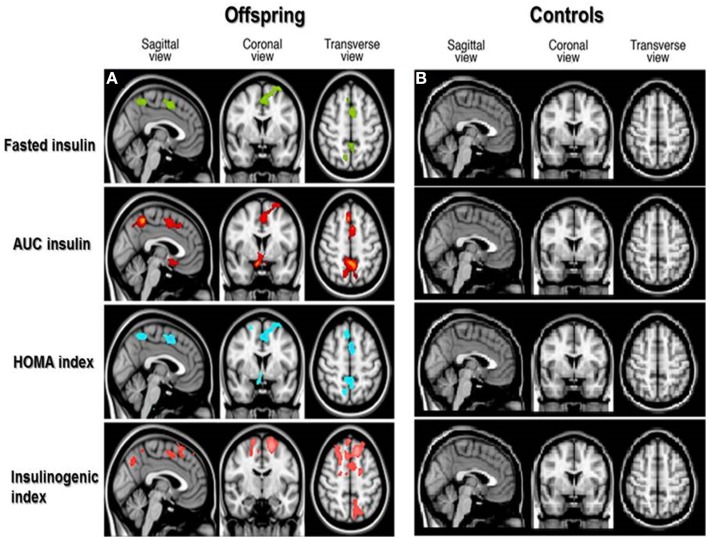
**Spatial distribution of associations between cortical gray matter MTR and insulin parameters in offspring and controls**. Voxel-based analysis of associations between cortical gray matter magnetization transfer ratio (MTR) and OGTT-derived insulin parameters in offspring **(A)** and controls **(B)**. Colored areas in brain slices show statistically significant associations between gray matter MTR and OGTT derived insulin parameters, with the different colors indicating significant associations as follows: fasted insulin in green, insulin Area Under the Curve (AUC) in red, Homeostatic Model Assessment (HOMA index) of insulin resistance in blue, and insulinogenic index in pink. No colored areas are seen in the controls, because the associations were not significant in the controls.

From VBM analysis, statistically significant associations between GM MTR were observed with different OGTT derived insulin parameters (*p* < 0.05) in the offspring, spatial distributions of these focal differences are shown in Figure 1. Similar significant associations were not observed for the controls.

### Chronological age: markers of glucose metabolism and microstructural brain parenchymal tissue homogeneity

**Figure 2:**
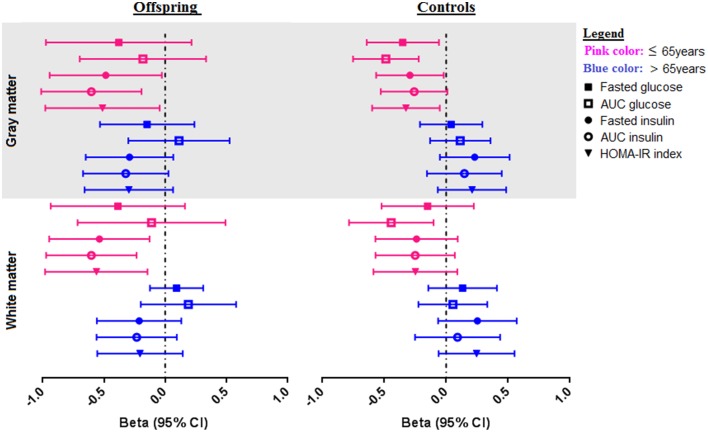
**Associations of OGTT parameters with gray and white matter MTR peak height in offspring and control groups, stratified for age**. Forest plots showing the distribution of the association of OGTT parameters (fasted glucose, AUC_glucose_, fasted insulin, AUC_insulin_, and HOMA-IR) with MTR peak height in gray and white matter in offspring and controls. The offspring and control groups were stratified into two age categories-≤ 65 years old (shown in pink color), and > 65 years (shown in blue color). Associations are from linear regression analysis, correcting for age and sex. Associations are presented as standardized Beta coefficients with corresponding 95% CI.

With the aim of investigating the effect of age on the association between parameters of glucose metabolism and MTR peak height, the offspring and controls were stratified based on age into two groups: subjects ≤ 65 years and > 65 years. The results are presented in Table 3 and Figure 2.

**Table 3:** **Association of MTR peak height with markers of glucose metabolism in “younger” and “older” offspring and controls**.

	**Offspring**					**Controls**				
	**≤ 65 years (*n* = 35)**		**> 65 years (*n* = 40)**		***P*-interaction**	**≤ 65 years (*n* = 30)**		**> 65 years (*n* = 27)**		***P*-interaction**
	**Beta**	***P*-value**	**Beta**	***P*-value**		**Beta**	***P*-value**	**Beta**	***P*-value**	
**GRAY MATTER**										
Fasted glucose	−0.379	0.203	−0.147	0.442	0.763	−0.350	**0.020**	−0.041	0.742	**0.033**
AUC glucose	−0.181	0.479	0.112	0.584	0.340	−0.487	**0.001**	0.114	0.341	**0.002**
Fasted insulin	−0.485	**0.038**	−0.291	0.107	0.567	−0.293	**0.037**	0.231	0.104	**0.014**
AUC insulin	−0.603	**0.005**	−0.322	0.069	0.293	−0.259	0.059	0.147	0.323	**0.042**
HOMA-IR	−0.512	**0.033**	−0.297	0.104	0.555	−0.325	**0.022**	0.209	0.129	**0.009**
Insulinogenic Index	−0.381	**0.007**	−0.442	**0.014**	0.623	0.003	0.986	−0.162	0.288	0.695
										
**WHITE MATTER**										
Fasted glucose	−0.386	0.160	−0.025	0.893	0.628	−0.149	0.418	0.133	0.330	0.128
AUC glucose	−0.333	0.154	0.190	0.327	0.072	−0.444	**0.013**	0.055	0.686	**0.033**
Fasted insulin	−0.537	**0.012**	−0.213	0.218	0.362	−0.239	0.152	0.253	0.113	**0.047**
AUC insulin	−0.603	**0.002**	−0.239	0.158	0.150	−0.250	0.120	0.092	0.584	0.121
HOMA-IR	−0.562	**0.010**	−0.206	0.239	0.351	−0.248	0.146	0.246	0.110	**0.038**
Insulinogenic index	−0.289	**0.032**	−0.360	**0.037**	0.557	0.124	0.601	−0.111	0.518	0.704

*All insulin values were log-transformed. Analyses were adjusted for age and sex, and associations are presented as standardized Beta coefficients (per increase in SD) with corresponding P-values*.

*The bold values represent significant results (p-values < 0.05)*.

Amongst the offspring, there were 35 subjects in the younger group (≤ 65 years), with mean age 61.3 (SD 3.2), age range 52–65 years. The older offspring consisted of 40 subjects with mean age 71.2 (SD 3.9), age range 66–84 years. In the younger offspring, decreased MTR peak height in cortical GM was significantly associated with higher fasted insulin (*p* = 0.038), AUC_insulin_ (*p* = 0.005), HOMA-IR (*p* = 0.033) and insulinogenic-index (*p* = 0.007). Likewise, in the white matter, decreased MTR peak height was significantly associated with OGTT derived measures of insulin resistance, namely, fasted insulin (*p* = 0.012), AUC_insulin_ (*p* = 0.002), HOMA-IR (*p* = 0.010), and insulinogenic-index (*p* = 0.032). Thus, in the younger offspring, parameters of reduced insulin action were significantly associated with decreased microstructural brain parenchymal homogeneity in both gray and white matter. Similar trends were seen in the older offspring (> 65 years), but the effects were smaller and did not reach statistical significance.

Of the controls, there were 30 subjects in the younger control group, with mean age 60.4 (SD 4.6) years, and age range 49–65 years. The older controls consisted of 27 subjects, with mean age 71.7 (SD 4.5) years, age range 66–82 years. In the younger controls, higher fasted glucose (*p* = 0.020), AUC_glucose_(*p* = 0.001), fasted insulin (*p* = 0.037), and HOMA-IR (*p* = 0.022) were associated decreased MTR peak height in cortical GM. Similar trends were seen in the white matter, but these did not reach statistical significance, except for AUC_glucose_ (*p* = 0.013). In contrast, in the older controls, no significant associations were found between OGTT-derived parameters of glucose or insulin metabolism and MTR peak height in gray or white matter.

From the comparison of the age categories in offspring and controls, although the association between OGTT parameters and gray and white matter integrity were present in the offspring and controls ≤ 65 years, the differences in the strength of the observed associations between age categories were only significant in the controls, as indicated by the *P*_interaction_ in Table 3. A visual representation of the associations according to age categories is shown in Figure 2.

## Sensitivity analyses

The aforementioned associations in the offspring, controls, and in both age groups of offspring and controls did not materially change after adjustment for age, gender, descent, smoking status, BMI and use of anti-hypertensive drugs. In addition, since there was a significant difference between the offspring and controls in the use of lipid lowering drugs, all the analyses were repeated with adjustment for use of lipid lowering drugs. The results did not materially change. In the offspring group, decreased GM MTR peak height, an index of microstructural brain parenchymal homogeneity was significantly associated with higher indices of insulin resistance (fasted insulin, AUC_insulin_ and HOMA-IR, all *p* < 0.01, see Table 2). After adjusting for use of lipid lowering drugs, decreased GM MTR peak height was still significantly associated with higher indices of insulin resistance (fasted insulin (β = −0.411, *p* = 0.018), AUC_insulin_(β = −0.415, *p* = 0.009), HOMA-IR (β = −0.436, *p* = 0.013), and insulinogenic index (β = −0.410, *p* = 0.012). Conversely, in the controls, GM MTR peak height was not significantly associated with indices of decreased insulin action, neither before nor after adjustment. Similarly, adjustment for lipid lowering medication did not materially change any of the results for white matter (Table 2) in offspring or controls (data not shown).

## Discussion

We report two main findings: Firstly, parameters of reduced peripheral insulin action were associated with reduced microstructural brain parenchymal homogeneity in the offspring group, but associations were less strong and did not reach statistical significance in the control group. Secondly, OGTT derived parameters of glucose metabolism were associated with reduced microstructural brain parenchymal homogeneity in “younger” older adults, but associations seemed less strong in older age groups. Thus, the associations between reduced insulin action and reduced microstructural brain parenchymal homogeneity seemed to dampen with age and to be stronger in familial longevity.

Previous studies have shown inverse associations between peripheral insulin action and brain structure and function. Inverse associations between insulin parameters (fasted insulin, HOMA-IR) and brain volumes as well as executive brain function and memory were reported in healthy older participants of the Framingham study (Benedetti et al., 2006). Furthermore, in another cohort of non-demented older adults, higher insulin levels (fasted insulin and AUC_insulin_) and impairments in insulin sensitivity were found to be associated with increased rate of cognitive decline (Burns et al., 2012). In line with these studies, we found an inverse association between parameters of peripheral insulin action (fasted and AUC_insulin_, HOMA-IR and insulinogenic index) and brain microstructural integrity.

Changes in the direction and strength of associations with age have previously been observed between other metabolic risk factors and cognitive decline. While high blood pressure, high cholesterol, and overweight are associated with cognitive decline and risk of dementia in (advanced) middle-age (Kivipelto et al., 2001; Yaffe et al., 2004; Whitmer et al., 2005), reversed associations were observed in very old persons, in which high blood pressure, high cholesterol and being overweight seem to protect against cognitive decline (Weverling-Rijnsburger et al., 1997; Elias et al., 2003). In our study population, we observed a weakening in the association between reduced insulin action and microstructural brain parenchymal homogeneity in advanced middle-age. Notably, in our study, we used MTI, which has the capacity of detecting microstructural changes in the aging brain. The aging brain is characterized by decline in total and segmental brain volumes, shrinkage of GM, loss of white matter, nerve fibers (axons), myelin and cells (Meier-Ruge et al., 1992). These subtle changes in microstructural brain parenchymal homogeneity, even in brain tissue that appears normal on conventional MR imaging sequences, can be detected and quantified using magnetization transfer imaging (MTI) (Filippi and Rocca, 2007). MTI is a sensitive MRI technique that is based on the exchange of magnetization between protons bound to macromolecules and protons of free water molecules inside tissue. The scale of this exchange is reflected in the magnetization transfer ratio (MTR) and peak height. The MTR peak height is specifically a measure of microstructural brain parenchymal tissue homogeneity (Rademacher et al., 1999) that is sensitive to age-related and disease-related brain parenchymal abnormalities (Benedetti et al., 2006; Sala et al., 2014), with a lower MTR peak height reflecting loss of homogeneity of the brain tissue, demyelination and axonal loss (Filippi and Rocca, 2007).

Due to the cross-sectional nature of our study, we cannot make any causal inference. Theoretically however, there are three possible interpretations for the inverse association between parameters of glucose metabolism and microstructural brain parenchymal homogeneity (brain integrity). The first theoretical explanation is that loss of brain integrity is a consequence of defects in glucose metabolism. T2D, characterized by hyperglycemia, insulin resistance and hyper-insulinemia, has been proposed to be involved in the pathogenesis of neurodegenerative diseases (Ott et al., 1999; Luchsinger et al., 2004; de la Monte and Wands, 2005), hallmark of which are progressive loss of nerve fibers and cells. This may be due to direct damage to the brain from high circulating glucose levels. It may also be due to secondary effects, including peripheral insulin resistance. A second theoretical possibility is that defects in glucose metabolism are a consequence of deficits in brain integrity. Emerging data from animal studies that show that the brain plays a physiologic role in glucose regulation (Lin et al., 2004; Lu et al., 2012; Morton et al., 2013; Schwartz et al., 2013) may support this possibility. The third possibility is that of the brain and metabolic dysregulation both being consequences of another common determinant. An example of a common pathway that may affect brain function as well as insulin resistance is oxidative stress (Jayaraman and Pike, 2014).

Several theoretical explanations exist for the observed differences in the associations between insulin action and microstructural brain parenchymal homogeneity with age and familial longevity. It is becoming clearer that the brain plays an important role in the regulation of peripheral glucose and insulin action [4]. Age related brain changes (reduced myelin and axons, and shrinkage of large neurons) are accompanied by reduction in brain volumes and function (Meier-Ruge et al., 1992). Brain control of glucose levels may also be affected, for which the body may compensate by higher peripheral insulin secretion. Our data show that higher insulin parameters are associated with decreased myelin and axonal integrity, and these are more pronounced in offspring and “younger” older adults in whom glucose-regulatory compensatory mechanisms are probably more intact. Another hypothetical possibility is that in the elderly and controls, diseases may be more prevalent that could reverse the association between insulin action and microstructural brain parenchymal homogeneity. For example, diseases that are associated with weight loss might improve insulin sensitivity but decrease microstructural brain parenchymal homogeneity. Systemic diseases such as chronic kidney disease, chronic respiratory disease, diabetes mellitus, and malignancies are more prevalent in the older adults, and may cause weight loss, which in theory would be associated with improved insulin sensitivity. However, since these are systemic illnesses, the disease itself may also decrease the integrity of the brain. For example, chronic kidney disease is associated not only with weight loss, but also with microvascular damage in the brain (O'Rourke and Safar, 2005).

This study is limited by its cross-sectional, observational nature. As such, the findings are purely correlative and descriptive. To clarify what is cause or consequence in the relation between insulin parameters and reduced brain integrity, intervention studies are required where insulin is specifically targeted to the brain (via the intranasal route) (Chapman et al., 2013), in humans of different age categories. Another limitation is that although offspring of long-lived families are included, not all of them would carry the longevity phenotype, leading to dilution of the observed results.

Strengths of this study include the use of sensitive MRI techniques, which have the discriminatory power to detect *in vivo* microstructural brain changes even when peripheral glucose and insulin levels are still within normal ranges. Another strength is its unique design of investigating the relation of parameters of glucose regulation and microstructural brain parenchymal tissue homogeneity from two contrast points-familial longevity and age. The incorporation of contrasts based on familial longevity with the use of offspring and their partners reduces the potential influence of environment, since the offspring share similar lifestyle and similar socio-economic and geographic background with their partners (age-matched controls), and so are highly comparable.

## Author contributions

AA designed the study, analyzed and interpreted the data, and wrote the report. AB co-analyzed the data, made the MRI figures and contributed to the intellectual content of the report. MB, SJ, ES collected the data and critically appraised, and edited the report. RW collected and interpreted the data, and critically appraised the report. JG and DH designed the study, analyzed and interpreted the data and contributed to the intellectual content of the report. All the authors edited and approved the manuscript before submission.

### Conflict of interest statement

The authors declare that the research was conducted in the absence of any commercial or financial relationships that could be construed as a potential conflict of interest.

## Abbreviations

AUC: Area Under the Curve
FMRIB: Functional MRI of the brain
GM: Gray Matter
HOMA-IR: Homeostatic Model Assessment of Insulin Resistance
LLS: Leiden Longevity Study
MR: Magnetic resonance
MRI: Magnetic Resonance Imaging
MTI: Magnetization Transfer Imaging
MTR: Magnetization Transfer Ratio
OGTT: Oral Glucose Tolerance Test
T2D: Type 2 Diabetes Mellitus
WM: White Matter.
