# Supplementary material for: Associations between insulin action and integrity of brain microstructure differ with familial longevity and with age
Source: Front Aging Neurosci. 2015 May 28;7:92. doi: 10.3389/fnagi.2015.00092 (PMC4446544; doi:10.3389/fnagi.2015.00092)
Supplement: Supplementary file 1 [file Table1.PDF]

Supplementary table S1. Correlation between cognitive tests and gray and white matter MTR peak height in groups of participants.

	Gray matter						White matter					
	**Whole group**		**Offspring**		**Partners**		**Whole group**		**Offspring**		**Partners**	
	r	*P* - value	r	*P* - value	r	*P* - value	r	*P* - value	r	*P*- value	r	*P* - value
**Cognitive tests**												
DSST	0.042	0.667	0.043	0.739	0.038	0.804	0.043	0.660	0.031	0.813	0.060	0.693
Stroop test	-0.091	0.314	-0.094	0.436	-0.092	0.510	-0.098	0.277	-0.035	0.770	-0.205	0.137
15-PLTi	0.112	0.216	0.172	0.151	0.025	0.859	0.121	0.180	0.198	0.098	0.023	0.871
15-PLTd	0.103	0.254	0.124	0.304	0.043	0.762	0.086	0.340	0.130	0.279	-0.005	0.971

Gray and white matter MTR histogram peak heights were derived from magnetization transfer MRI, and used as a measure of microstructural brain parenchymal tissue homogeneity.
Associations are from bivariate Pearson correlation analysis.
r: Pearson correlation coefficient; DSST: Digit Symbol Substitution Test; 15- PLTi: 15- Picture Word Learning Test immediate recall; 15- PLTd: 15- Picture Word Learning Test delayed recall.
